# Supplementary material for: Does GPA matter for university graduates’ wages? New evidence revisited
Source: PLoS One. 2022 Apr 12;17(4):e0266981. doi: 10.1371/journal.pone.0266981 (PMC9004755; doi:10.1371/journal.pone.0266981)
Supplement: S1 Appendix — (DOCX) [file pone.0266981.s001.docx]

**S1 Appendix: The unconditional quantile regression**

Unconditional quantile regression is a special case of recentred influence function (RIF) regression [1]. The RIF is defined as

$\mathrm{RIF}\left( y;v(F_{Y}), F_{Y} \right)=v\left( F_{Y} \right)+IF\left( y; v(F_{Y}), F_{Y} \right),$ (A.1)

where $v\left( F_{Y} \right)$ is a distributional statistic of interest, and $\mathrm{IF}\left( y; v(F_{Y}), F_{Y} \right)$ is the influence function of this specific distributional statistic. The influence function $\mathrm{IF}\left( y; v(F_{Y}), F_{Y} \right)$ of $v\left( F_{Y} \right)$ represents the influence of an individual observation on that statistic. Adding back the statistic $v\left( F_{Y} \right)$ to the influence function yields the so-called recentred influence function (RIF). A convention feature of the RIF is that its expectation is equal to $v\left( F_{Y} \right)$. One of the most important strengths of the above expression is that it “can be used directly for the estimation of standard errors of any statistic for which a RIF exists [2]”.

The RIF regression uses the estimated $\mathrm{RIF}\left( y;v(F_{Y}), F_{Y} \right)$ for each observation $y_{i}$ in the data as the dependent variable and regresses it against all the variables of interest:

$\mathrm{RIF}\left( y;v(F_{Y}), F_{Y} \right)=X\cdot\varphi+\varepsilon, E(\varepsilon)=0.$ (A.2)

When $v\left( F_{Y} \right)$ is the quantile, the RIF regression becomes the unconditional quantile regression. The coefficient $\varphi$ gauges the impact of the change in the distribution of an explanatory variable on the marginal distribution of $Y$.

**Reference**

- - - 1. Firpo S, Fortin NM, Lemieux T. Unconditional quantile regressions. Econometrica. 2009; 77(3): 953-973.
      2. Rios-Avila F. Recentered influence functions (RIFs) in Stata: RIF regression and RIF decomposition. The Stata Journal. 2020; 20:51 - 94.
