# Supplementary material for: Does GPA matter for university graduates’ wages? New evidence revisited
Source: PLoS One. 2022 Apr 12;17(4):e0266981. doi: 10.1371/journal.pone.0266981 (PMC9004755; doi:10.1371/journal.pone.0266981)
Supplement: Attachment — Submitted filename: Response.docx [file pone.0266981.s003.docx]

Dear editor

Thank you very much for your prompt handling of my paper. I apologize for my previous misunderstanding and uploading the incorrect ethical review approval document. I am writing to clarify the process of our ethical review and upload the initial ethical review approval document and hope I would still have a chance to re-submit our manuscript to PLOS ONE. Our detailed response is following your comments (italic font). Thanks, and best wishes.

*Dear Dr. Zou,*

*Thank you very much for submitting your manuscript to PLOS ONE. After careful consideration, we have decided that your manuscript does not meet our publication criteria and thus, cannot be considered further for publication.*

*PLOS ONE's publication criteria require that research must have been conducted to the highest ethical standards and must meet all applicable standards for the ethics of experimentation and research integrity (http://journals.plos.org/plosone/s/criteria-for-publication#loc-6). As an international journal, we reserve the right to reject work, even when authors may have followed local laws and guidelines.*

*We understand that you obtained ethical approval from your local board after you began these experiments: your ethics approval document is dated Jan 12, 2022, after your studies were conducted. PLOS ONE requires that authors obtain ethics committee approval before beginning the study; we do not accept retrospective ethical approval.*

**Response**: The fact is that we have gone through several ethical reviews for each of our related research projects. At our university (SWUFE), the proposal, outline, questionnaire, research output, etc., would be examined by the ethical review committee to make sure no confidential information is disclosed and no ethic rules are violated. The research, on which our current manuscript is based, has been reviewed and approved twice: one is before I started my survey and the other one is when I began to write a paper, which were in 2018 and 2020, respectively. Regarding the retrospective ethical approval, you mentioned, I have to clarify that I misunderstood it — I had thought you were asking for a new certification and I uploaded only the recent approval which screens only research output in the paper. I did obtain the two copies of the original ethics review approval provided by the Ethics Committee of Career Guidance Center of SWUFE (Southwestern University of Finance and Economics). I am now attaching both the 2018 and the 2020 copies for your reference.

*For the above reasons, we are rejecting your manuscript. I am sorry that we cannot be more positive on this occasion, but hope that you understand the reasons for this decision.*

*Sincerely,*

*Yann Benetreau, PhD*

*Division Editor, PLOS ONE*
